# The Challenge of Rebuilding Gaza’s Health System: A Narrative Review Towards Sustainability

**DOI:** 10.3390/healthcare13151860

**Published:** 2025-07-30

**Authors:** Eduardo Missoni, Kasturi Sen

**Affiliations:** 1 Master of International Healthcare Management Economics and Policy (MIHMEP), SDA Bocconi Management School, 20136 Milan, Italy; 2 Wolfson College, University of Oxford (CR) United Kingdom, Oxford OX2 6UD, UK

**Keywords:** Gaza Health System, health infrastructure, Gaza conflict, sustainability and population health

## Abstract

**Background**: Since the election of Hamas in 2006, Gaza has endured eight major military conflicts, culminating in the ongoing 2023–2025 war, now surpassing 520 days. This protracted violence, compounded by a 17-year blockade, has resulted in the near-total collapse of Gaza’s health system. Over 49,000 deaths, widespread displacement, and the destruction of more than 60% of health infrastructure have overwhelmed both local capacity and international humanitarian response. **Objectives**: This narrative review aims to examine and synthesize the current literature (October 2023–April 2025) on the health crisis in Gaza, with a specific focus on identifying key themes and knowledge gaps relevant to rebuilding a sustainable health system. The review also seeks to outline strategic pathways for recovery in the context of ongoing conflict and systemic deprivation. **Methods**: Given the urgency and limitations of empirical data from conflict zones, a narrative review approach was adopted. Fifty-two sources—including peer-reviewed articles, editorials, reports, and correspondence—were selected through targeted searches using Medline and Google Scholar. The analysis was framed within a public health and political economy perspective, also taking health system building blocks into consideration. **Results**: The reviewed literature emphasizes emergency needs: trauma care, infectious disease control, and supply chain restoration. Innovations such as mobile clinics and telemedicine offer interim solutions. Gaps include limited attention to mental health (including that of health workers), local governance, and sustainable planning frameworks. **Conclusions**: Sustainable reconstruction requires a durable ceasefire; international stewardship aligned with local ownership; and a phased, equity-driven strategy emphasizing primary care, mental health, trauma management, and community engagement.

## 1. Introduction

Since Hamas was elected to power in Gaza in 2006, there were eight wars which occurred between 2008 and 2023, alongside a total blockade that made daily survival a major challenge. The 2008 war lasted 23 days, 2012 for 8 days, 2014 for 50 days, and 2018 involved a mass shooting at the northern border, killing and injuring hundreds protesting the blockade [1]. The 2021 war lasted 11 days. Now we are into the eighth war. Each war caused deaths, injuries, and destruction of health and residential infrastructure. The 2023 war has to date lasted over 520 days, with near-total devastation of lives, health infrastructure, and the environment. The toll is a staggering one [2,3], leading to many agencies, including the UN, describing the situation as a genocide [4].

Accurate figures are difficult to obtain in most conflict zones, but estimates suggest over 49,000 killed by the end of March 2025, mostly women and children (70%), 110,000 injured, and 30,000 missing. Over 80% of residences have been destroyed, with 60% of hospitals non-functional and the rest barely operational [5]. Severe shortages of medicines and oxygen persist [2]. UN agencies, including the Office for Coordination of Humanitarian Assistance (OCHA), the United Nations Relief and Works Agency for Palestine (UNRWA), and the World Health Organization (WHO), provide weekly updates, documenting the humanitarian crisis and the immense challenge of rebuilding Gaza [2,6,7,8,9].

Rebuilding Gaza’s health system must consider the ongoing conflict, which causes severe attrition at individual, collective, and environmental levels. Each war worsens mortality, morbidity, infrastructure loss, and environmental destruction. With increasing war intensity and advanced weaponry, deaths and damage have escalated. The conflict continuum shows recurring health system challenges across decades.

The aim of this narrative review is to describe the key issues raised in the literature to date (October 2023–April 2025) on rebuilding the health system of Gaza, in the most sustainable way. Whilst the overall scenario of planning a rebuild in the midst of conflict is a challenge, the narrative aims to extract information and highlight the possibilities.

Recent literature (past 18 months) includes editorials, correspondence, and short reports, reflecting urgent publication needs. Empirical research is limited due to on-ground conditions. However, studies from 2015 to 2024 by Gaza-based scientists offer important historical insights into Gaza’s population and health system. These include an online cross-sectional study on medical needs; a longitudinal study on siege impacts; a self-administered prevalence study on displaced populations’ mental health; a patient case study on the use of telemedicine to support trauma surgery; and a qualitative study based on Focus Group Discussions (FGDs) with healthcare professionals, humanitarian workers, and members of the community [10,11,12]. The only longitudinal study undertaken in Gaza over several years [11] is included, as it provides an invaluable link between past and present.

In the methodology section below, we describe the criteria followed to elaborate on the narrative. Findings are structured into the two following sections: “Themes” and “Gaps”. These illustrate the different aspects of the challenge of rebuilding a sustainable health system and the current limitations of existing literature. Finally, we conclude by summarizing our thoughts about the “Way forward”.

## 2. Methodology and Framework

In any situation, health system sustainability and universal access depend on diverse determinants and intersectoral collaboration, incorporating health priorities into all public policies [13]. In conflict or post-conflict conditions, local ownership, primary prevention, and appropriate care are even more critical. With this approach in mind, our Narrative Review explores the feasibility and challenges of rebuilding the health system of Gaza sustainably. In reflecting on the future, wherever possible, we make reference to the different components or “building blocks” of the healthcare system (leadership and governance; human resources; medical products and technology; financing; service delivery; and information) [14,15].

The exploratory nature of our research question, which asks whether the health system can be rebuilt in the aftermath of a devastating conflict; the small number of papers, comments, and correspondence available; and the time span (18 months) did not allow for a systematic review. Instead, we focused our methodology on a ‘state-of-the-art’ narrative review, summarizing the research on the topic along the timeline and identifying the gaps in content that allow us to better assess the question of rebuilding the health system of Gaza in a sustainable way. Indeed, a narrative review is a well-established method allowing for flexibility in a context such as that of Gaza with ever-changing political, geographical, and temporal boundaries [16]. The chosen framework also allowed us to address our research question from the perspective of a diverse range of disciplines under the rubric of public health, including the social sciences and humanities [17].

This review is a summary of the current status of thinking on the topic, how the situation has evolved since the onset of the conflict in 2023 until our submission in early April 2025, and what direction it may take for rebuilding a sustainable health system in Gaza. The papers we describe and analyze were selected using Google Scholar and Medline as search engines following the keywords: Gaza Health System, Health infrastructure, Gaza Conflict, Sustainability and Population health. All selected papers were in English. A majority of the literature included covered the impact of the conflict. A core issue was to understand the factors affecting the collapse of the health system and infrastructure and to explore possibilities for its future. The time span was an important criterion for exclusion in order to focus on the current conflict. We did not include an exclusive analysis of mortality and morbidity because there was already a reference to this in the majority of reports and papers reviewed.

Selected reports and papers were read in depth to extract *themes* that would help generate a narrative for the rebuilding of Gaza’s health system and identify knowledge *gaps* that need to be addressed. For those covering the health system, conflict, health infrastructure, and population health, 52 papers, including published reports, editorials, analyses, comments, and correspondence, were included between October 2023 and April 2025 (Table 1).

There were a small handful of empirical studies, mostly from physicians working on site, but generally, fieldwork is not feasible in an active war zone.

Papers that covered issues for Palestine as a whole, including Gaza, or those that did not address our keywords, were excluded. The review is organized in a manner that provides a thematic synthesis of the context relating to the state of the health system of Gaza.

## 3. Results

### 3.1. Themes

Selected literature assesses the damage and addresses immediate needs. They are mostly descriptive and data-driven papers that prioritize rapid data collection over in-depth analysis in a dynamic crisis. Compiled by multilateral aid organizations closest on the ground, reports document deaths, injuries, and infrastructure loss in collaboration with Gaza’s Ministry of Health. The reports highlight severe shortages of medicines, oxygen, and equipment while condemning attacks on health workers and facilities. All agencies call for an urgent ceasefire and the resumption of humanitarian aid [2,6,7,8,9,18,19].

Established in 1948 to support Palestinian refugees, UNRWA regularly produced Situation Reports on the humanitarian emergency, covering food, shelter, and healthcare [20]. It also commissioned thematic reports on public health, such as highlighting the urgent need for psychosocial and mental health support (MHPSS) in Gaza. A report from the first year of conflict by UNRWA details how over 300 days of war have deepened the mental health crisis, causing severe, lasting effects that will require a long-term MHPSS response. Restricted humanitarian access, including to mental health services and to medication, it argues, worsens suffering, especially for those with pre-existing psychiatric conditions [20]. Reports from these agencies provide key insights into the immediate and long-term health and welfare needs of Gazans, covering social determinants like shelter and education [7]. Situation reports also call for immediate access to aid and to enable emergency planning for affected families. They focus on addressing urgent humanitarian needs rather than the long-term rebuilding of the health system. WHO reports highlight the immense challenge of rebuilding Gaza’s health system due to political instability, the loss of health workers, and acute resource shortages. Like WHO, the ICRC reports emphasize the urgent need for access to humanitarian aid [7,9].

Multilateral agencies face two key concerns: First and foremost, ensuring a durable ceasefire, as past ones witnessed the targeted killings of aid workers. They are also concerned with the immediate need for massively upgrading supply chains for food, fuel, medicine, and water, which the war has severely depleted [9]. In addition to reports, several UN agencies have published correspondence in major journals, highlighting issues raised in their day-to-day work from attacks on health infrastructure and the killing of health workers [21]. They highlight the devastating impact of the conflict on children and pregnant women, coupled with the impossibility of working without a humanitarian ceasefire and all supplies cut off [22]. Despite their joint efforts, including a public statement early on in the conflict in November 2023, urging for a humanitarian ceasefire, the conflict continued unabated [23,24].

A major challenge for agencies is the ability to coordinate humanitarian efforts. While health clusters exist throughout the OPTs and Gaza, scarce resources and political and conflict constraints can often lead to siloed work. Given the extreme shortages, pooling financial and human resources across sectors may be necessary. Nearly 300 UN/WHO health workers have been killed to date, with many more injured [25]. Situation reports highlight little opportunity for collaboration with local communities who have been enduring extreme injury, death, and starvation. The priorities of communities will vary based on location, damage, and personal loss.

Smith argues that Gaza’s siege, an overlooked geopolitical process, undermines healthcare through supply chain control and systemic destabilization. His qualitative interviews suggest that siege deliberately renders the population dependent, as surplus, and captive, preventing a functional health system. Gaza’s infrastructure has been continually stripped, leaving residents reliant on Israel, international bodies, and aid [11]. Despite the siege, Al Ashawwaf et al. highlight local efforts to counteract the health system’s collapse [10].

Medical Points (MPs), akin to rudimentary mobile clinics, operate in 28 areas, providing primary care and trauma management for displaced populations. Even though these have been systematically shelled, MPs have persisted despite severe shortages of medication, equipment, and staff due to supply chain control and health worker casualties [10]. Replacing health workers remains a major challenge, jeopardizing vulnerable populations, increasing preventable deaths, and hindering responses to acute and chronic health needs. Al Ashawwaf et al. advocate for innovative solutions such as telemedicine and expanding mobile clinics. They call for urgent international action to ensure sustainable emergency healthcare [10]. While Alshawwaf et al. focus on MPs [10], Musa et al. document nine years of conflict in Gaza in a *Lancet* letter. They compare the 2014 war to the ongoing 2023 conflict, highlighting the relentless destruction by Israel [26].

By November 2023, two-thirds of Gaza’s hospitals and half of UNRWA-run educational facilities—used as shelters—were destroyed. Ambulances transporting critically ill patients to Egypt were also disabled by shelling. By February 2024, it was reported that “every hospital in Gaza is either damaged, destroyed, or out of service due to lack of fuel” [27].

Musa et al. report that six weeks into the conflict, Gaza’s death toll since 7 October 2023 exceeded 11,000, with over a third of them being children. More than 27,000 were injured. As Israeli military operations targeted the north and Gaza City, 70% of Gaza’s 2.2 million residents were forced to the south, yet even those fleeing or already displaced were not spared from being targeted. They criticize the international medical community’s silence on Gaza’s crisis, arguing that the medical community has a moral duty to speak out for an end to the conflict. They highlight violations of International Humanitarian Law protecting health workers and infrastructure that has been totally ignored by Israel. Like other reports, they stress that only a durable ceasefire can enable sustainable rebuilding. They call for a political process and the urgent enforcement of international laws [26].

Zayed et al. [28] and Dardona et al. [29] highlight the rapid spread of infectious diseases and the long-term consequences of leaving them untreated due to the targeted destruction of water, sanitation, and electricity infrastructure. Diseases spreading rapidly, particularly from repeated displacement and lack of shelter, include scabies, head lice, chickenpox, diarrheal diseases, and hepatitis. Recently published data highlight the staggering numbers of cases. Since the inception of the conflict, there have been 40,000 recorded cases of hepatitis, nearly one million acute respiratory tract infections, half a million cases of acute diarrhea, and over 10,000 cases of jaundice [30]. In a densely packed area and with the population repeatedly displaced, these figures are likely to be underestimates. The lack of medical supplies and equipment means many infections will remain untreated and create further health complications, as highlighted by Irfan and 22 other medical mission authors in Gaza who discuss the challenges of infection control under a siege. They argue that relentless shelling, bombing, and orders from Israel to halt health work or evacuate health premises prevent patients from accessing much-needed care and health workers from providing it [31].

Of particular concern in the literature is the re-emergence of polio, previously declared eradicated by Gaza’s Ministry of Health since 1999. This has been exacerbated by the collapse of the vaccination programs and especially by the loss of the sanitation system, with polio detected in sewage early on in the conflict [29]. Several authors call for immediate action to prevent lifelong disabilities in children and for the supply of equipment to detect and prevent further spread of the virus. They emphasize the need to protect mobile clinics (MPs) despite limited resources to undertake such work. The WHO’s emergency vaccination program, launched in July 2024, targeted 600,000 children under a temporary ceasefire [28]. However, according to the WHO, the ongoing repeat displacement hinders efforts to control polio. They estimated that some 7000–10,000 children in hard-to-reach areas were at risk of long-term paralysis and disability [7]. Hamdollahzadeh and Karami [32] and Balkhy and Ghebreyesus [25] emphasize that preventing long-term disabilities, such as amputations, will be crucial for rebuilding Gaza’s health system.

The latest onslaught has left a majority of hospitals, health clinics, and primary healthcare centers without the ability to function effectively against overwhelming need, as highlighted in many of the papers. Mahmoud and Abuzerr cite the MOH of Palestine on the systematic obstruction of Gaza’s health system during the 17-year siege, worsened by the current conflict. Repeated attacks left the health infrastructure fragmented, lacking sub-specialist services, and unable to import essential medical equipment. Prior to October 2023, only 35 hospitals with 3412 beds served over 2 million people [33].

The crippled health system in Gaza has also severely impacted those with chronic conditions, such as diabetes and heart disease, which require ongoing care and medication. At the beginning of the current conflict, some 350,000 people suffered from chronic conditions, including cancer, diabetes, chronic renal failure, and heart failure, while nearly 50,000 pregnant women lacked essential care. The need for a continuous supply of medicines and equipment will be a major factor in preventable morbidity and mortality in the future. The inability to provide vital medications, along with fuel shortages and lack of electricity, will simply lead to more deaths. Almost 95% of the population of Gaza lack clean water, and over 85% live in poverty. Since 7 October, Israel’s complete embargo has cut off fuel, power, food, and clean water [34,35].

Al Khalidi and Airubai offer a perspective on rebuilding Gaza’s health system, suggesting that despite the overwhelming crisis, a fundamental rethinking could address immediate, medium, and long-term needs in phases. The proposed phases are based on an inclusive national approach to health services across Palestine. Their ‘crisis recovery plan’ mirrors a post-WWII Marshall Plan for Germany, with international, regional, and national collaboration for planning and financing. The immediate priority is a comprehensive health sector needs assessment: (1) immediate needs assessment, humanitarian aid, and medical support, (2) rebuilding people’s survival and livability, and (3) reconstructing public systems, including the health sector. This plan may last at least 10 years, with Gaza’s healthcare system reconstruction aligned with WHO standards and integrated into broader national recovery efforts. However, a permanent ceasefire is essential for any action [36].

This call for a ceasefire is also supported by Al Alokaily in the *Saudi Medical Journal*, who argues that the conflict has created a prolonged public health emergency due to a lack of access to essential life-saving needs, including medical supplies and quality care. While all of the population is affected, in this view, children are the most severely impacted by trauma and displacement. Al Alokaily urges Saudi Arabia to apply regional political pressure on Israel to reopen supply chains and, once again, calls for a durable ceasefire [37].

A special issue of the *Eastern Mediterranean Health Journal (EMHJ)* on the Gaza war features articles on Gaza’s health system, including commentaries, reviews, and prevalence studies [38]. The papers detail war’s impact, from injury care to the loss of Gaza’s only psychiatric hospital. The articles provide evidence-based recommendations for urgent and long-term health system recovery. Like most reviewed papers, it calls for an urgent and lasting ceasefire. In the editorial, Balkhy and Ghebreyesus outline the Gaza conflict, emphasizing healthcare as its primary casualty. They present grim statistics on deaths, injuries, displacement, and the destruction of hospitals and PHCs. They also highlight the toll on health workers, with over 670 attacks on patients, medical staff, and ambulances by early 2025 [25]. Table 2 below highlights deaths and injuries to date.

Amid shortages and relentless bombardment, the WHO faces major challenges in delivering healthcare. It prioritizes immediate needs, addressing mental and physical trauma through mobile clinics and teleconsultations. The WHO also highlights the often-overlooked mental and physical well-being of healthcare workers, many severely traumatized. Like much of the literature cited, it stresses a permanent ceasefire as essential for health restoration [25].

Husseini from the Institute of Palestine Studies, Amman, provides a critical overview of Gaza’s public health system. Using a political economy approach, he defines public health as addressing both physical and mental well-being. He argues that the Occupation of Palestinian lands by Israel has denied Gaza all aspects of public health since the siege began 17 years ago. Husseini also argues that the siege, alongside a policy of impoverishment, has restricted work, trade, and manufacturing, which has severely impacted life in Gaza. Eight wars and invasions from 2008 to 2023 caused killings, destruction, and widespread violence, harming both physical and psychological health. He considers that Gaza’s public health must be understood through its social and political determinants, with the Occupation as being central to any health system resolution. Husseini emphasizes considering ‘quality of life’—beyond deaths and deprivation—including mental health as an integral part of any recovery. He concludes that restoring essentials for life in Gaza starts with addressing the Occupation, a long-term crisis that has been magnified by the current conflict [39].

### 3.2. Gaps

Most papers on Gaza’s health system focus on immediate needs—clean water, sanitation, and the reopening of supply chains for food, medicine, and oxygen. Nearly all call for safe spaces and a durable ceasefire, which remains unrealized. They collectively show that the scale of need is overwhelming. Many, including UN briefs, highlight the urgent need for mental health services, especially for injured, orphaned, and displaced children; however, this is not consistently addressed. Aqtam finds high mental health disorder rates due to ongoing violence and displacement, with gaps in care, as physical injuries take priority. However, few address the disproportionate mental health impact on women and on health workers [40]. Furthermore, support for traumatized health workers is a very important gap in the literature since there will be an explosion of need for healthcare in the foreseeable future with a severely depleted workforce [21,22].

Most papers overlook the essential role of place in security, which is vital for rebuilding Gaza’s health system. With limited health workers and finances, a stronger UN, NGO, and state collaboration is needed for a holistic recovery. Few papers emphasize assessing displaced and traumatized populations’ priorities through local community consultation. The exception is a case study of global telemedical support for medical teams on trauma surgery based at one Gaza hospital. It built a remarkable community of professionals through this intervention [41].

Despite enduring many wars, Gazans likely have local groups addressing their urgent needs through community collaboration and support. It appears that the current conflict aims to erase place, belonging, and memory more than any one previously. Collaborating with local organizations is key to sustainable rebuilding, prioritizing people to foster community ownership and future governance despite the challenges.

In an overwhelming crisis such as that of Gaza, there has been a flourishing number of papers covering the most urgent and immediate needs, as identified above. The gaps identified are important, but do not underestimate the challenges faced by medics, the multilateral agencies, and researchers on site to spread the message through reports and publications. This cannot be faulted in any way. The gaps we have identified may be incorporated into any plans if and when there is a durable ceasefire.

## 4. Conclusions: Ways Forward—The Future

The way forward for Gaza remains challenging. Ending the Occupation is essential for self-determination and freedom of movement. Stopping the war, withdrawing occupation forces, lifting the siege, and ensuring movement are prerequisites for any effective health intervention [39].

International law has failed to halt hostilities and restore humanitarian aid, both crucial for normalcy [3]. Global health actors have also struggled to address the crisis [42,43]. Stewardship and governance of the reconstruction process is a major challenge, and a coordinated international effort will be essential, with the World Health Organization (WHO) and other UN agencies playing a leading role, ensuring Palestinian authorities and the MOH of Gaza have full ownership of the process. Clear roles should be established at both the local and international levels to ensure alignment and harmonization of aid, avoid duplication, and maximize efficiency.

In terms of financing, a large-scale increase in international funding is needed to address immediate health needs and restore the health system, with aid delivery restrictions lifted for essential supplies [44]. A joint international fund would facilitate alignment and harmonization of aid, avoid duplication, and maximize efficiency. Unconditional financial, logistical, and expert support should focus on rebuilding healthcare infrastructure and creating a sustainable health system, not just addressing immediate needs [45]. Short-term aid cannot fix Gaza’s healthcare system’s long-standing weaknesses. To ensure equity in access to healthcare, the aim should be the development of a universalistic health system, avoiding out-of-pocket purchase of services. Efforts should focus on creating a fair, sustainable framework, reducing reliance on external support [46].

Population health management, a proactive approach for at-risk populations like Gaza, involves organizational, cultural, and individual interventions. Key elements include reorganizing care models, empowering communities, strengthening governance, integrating services, and creating a supportive environment [47]. In Gaza’s unprecedented humanitarian crisis, addressing basic needs like food, shelter, safety, water, and sanitation must align with service delivery and reorganizing the healthcare system [48]. A cooperative, intersectoral approach, endorsed by health authorities, is crucial, with over 60 partners to date providing healthcare services [47]. Coordinated interventions from international organizations, NGOs, local governments, and communities are essential for immediate and long-term responses [49]. The long-term impact of starvation policies, particularly for the most vulnerable, such as children, infants, and lactating mothers, requires intervention, such as emergency feeding programs, to address their serious health consequences [50].

The devastation from bombing also harms the environment, disrupting natural processes and jeopardizing the region’s sustainability, worsening the health crisis and increasing healthcare needs, with long-term ecological impacts [50,51]. With 80% of infrastructure destroyed, restoring water, sanitation, and natural habitats is critical to preventing further health and environmental crises [29]. Without functioning water, sanitation, and electricity, no sustainable health system can succeed. Sustainable resource management is crucial for preserving Gaza’s natural resources, including decentralized solar power, water purification, and waste management systems. Alongside infrastructure, educational initiatives should empower Gazans to adopt sustainable practices like recycling and composting [52]. All said, community involvement is essential in all stages, from needs assessment to implementation. It helps address overlooked needs, such as proper family burials, which may alleviate the huge trauma [25].

Rebuilding infrastructure is unlikely in the short term, but mobile clinics, which are already in place, need protection and an increase in numbers to address the physical and mental trauma, infectious diseases, and chronic conditions [52]. The focus should be on primary healthcare, emphasizing prevention, community services, and first-level care to reduce hospital burden and enhance system adaptability. Integrating culturally competent mental health care, once active, into primary services will be key for long-term recovery, alongside strengthening local medical education and workforce training [49].

Health information systems (HIS) are vital but challenging in conflict areas. In Gaza, most medical records have been destroyed, and hence, it will be essential to work out how best to restore some semblance of a HIS, including the management of basic epidemiological surveillance. Mobile technology can support data exchange, managerial functions, and care orientation [49]. The effectiveness of telemedical support is highlighted in the work of the global telemedicine initiative for trauma care under enormously challenging circumstances and with positive impacts [41].

Sustainability also requires local production of essential medicines and equipment. Rebuilding Gaza’s health system must be guided by a transformative vision, recognizing health as a political issue [46].

Mobile education systems implemented to address the educational deprivation of Gaza’s young population may also usefully convey supportive health education [53].

**Figure 1:**
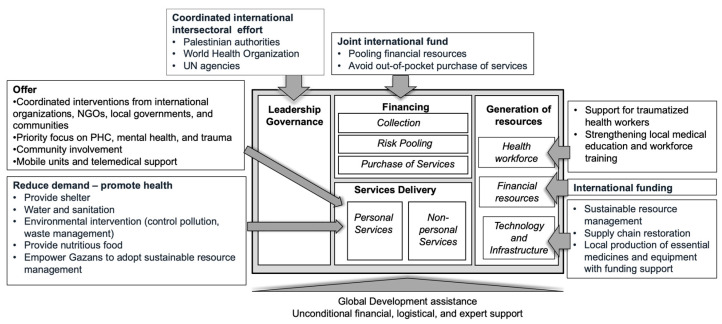
Supporting rebuilding interventions and health system building blocks (Source: compiled by authors, 2025).

Figure 1 below summarizes needed interventions in the framework of a health system’s ’building blocks’.

The ongoing conflict is an unprecedented one, which is now widely accepted as ethnic cleansing and genocide by Israel. It did not begin in October 2023, but several decades earlier for the whole region. There has been an intensification of it for over 17 years in Gaza, placed under a suffocating siege. Now, as this narrative highlights, much of Gaza has been reduced to rubble, the soil and water sources poisoned through intense bombing and destruction, together with unimaginable environmental catastrophe and human suffering. Accompanying this, the conflict has been a destruction of the laws of war, the Geneva Convention, and International Humanitarian Law on the protection of civilians and health workers. This places a grave challenge not just for Palestine or the Gaza Strip but for the world as a whole, further weakening the multilateral system established after WWII, dramatically increasing the risk of a new world conflict, and making the world an unsafe place for all of humanity.

The ceasefire of 19 January 2025 was broken in March, leading to extensive bombing and hundreds of civilian deaths, particularly of women and children. The conflict is ongoing, but our suggested Ways Forward remain. Humanitarian agencies and Gaza’s Ministry of Health must urgently restore basics like water, sanitation, food, and electricity. Once achieved, international pressure is needed to revive the supply chain for food, medicines, and medical equipment. An immediate priority is addressing trauma and injury through mobile clinics, along with rehabilitation centers and MHPSS services. However, little can be accomplished without a durable ceasefire.

## Figures and Tables

**Table 1:** Papers, reports, comments, and studies (excluding 3 studies under Methods).

Reports	Journal Articles	Editorials	Commentary	Correspondence	Policy Paper
12	21	5	5	8	1

**Table 2:** Gaza deaths and injuries by category (October 2023–June 2025).

Category	Total	Daily Average
Total Killed	54,981	89.69
Total Children Killed	18,000	29.36
Total Women Killed	12,400	20.23
Total Men Killed	24,581	40.1
Total Injured	126,920	207.05
Total Civilian Defense Killed	115	0.19
Total Medical Personnel Killed	1583	2.58
Total Press Killed	227	0.37

Source: Adapted from https://www.goodshepherdcollective.org/data/gaza_deaths (accessed on 22 June 2025).
